# Gendered Impacts of the COVID-19 Pandemic on Food Behaviors in North Africa: Cases of Egypt, Morocco, and Tunisia

**DOI:** 10.3390/ijerph19042192

**Published:** 2022-02-15

**Authors:** Tarek Ben Hassen, Hamid El Bilali, Mohammad S. Allahyari, Islam Mohamed Kamel, Hanen Ben Ismail, Hajer Debbabi, Khaled Sassi

**Affiliations:** 1 Program of Policy, Planning, and Development, Department of International Affairs, College of Arts and Sciences, Qatar University, Doha 2713, Qatar; thassen@qu.edu.qa; 2 International Centre for Advanced Mediterranean Agronomic Studies (CIHEAM-Bari), Via Ceglie 9, 70010 Valenzano (Bari), Italy; islam_hankhater@yahoo.com; 3 Department of Agricultural Management, Rasht Branch, Islamic Azad University, Rasht 41476-54919, Iran; allahyari@iaurasht.ac.ir; 4 Faculty of Economic and Management Sciences, North-West University, Private Bag X2046, Internal Box 575, Mmabatho 2735, South Africa; 5 Department of Agri-Food Industries, National Agronomic Institute of Tunisia, University of Carthage, Tunis 1082, Tunisia; hanene.benismail@inat.u-carthage.tn (H.B.I.); hajer.dabbabi@inat.u-carthage.tn (H.D.); 6 Department of Agronomy and Plant Biotechnology, National Agronomic Institute of Tunisia, University of Carthage, Tunis 1082, Tunisia; khaledsassi1@gmail.com

**Keywords:** COVID-19, SARS-CoV-2, food behavior, food shopping, food consumption, food waste, gender, Egypt, Morocco, Tunisia, North Africa

## Abstract

The COVID-19 pandemic-related measures in the Near East and North Africa (NENA) region have resulted in many lifestyle modifications, including changes in diet and food buying patterns among adults. However, the pandemic has impacted women and men differently and exacerbated existing socio-economic and gender inequalities. Indeed, numerous studies conducted worldwide have shown that the COVID-19 pandemic had a disproportionately negative impact on women compared to males. Therefore, this paper aims to analyze the effects of COVID-19 on women’s food behaviors in three countries of the North Africa sub-region, namely, Egypt, Morocco, and Tunisia. The study was based on an online poll conducted by SurveyMonkey from 15 September to 5 November 2020, with 995 participants. The outcomes of the research found that when compared to men, (1) women tend to consume more food out of fear, anxiety, or boredom; (2) women prefer to eat more unhealthy food; (3) women tend to stockpile a greater amount of food; and (4) women tend to modify their shopping habits more often. The findings should inform gender-sensitive strategies and policies to address the negative impacts of the pandemic and foster transition towards healthier diets and resilient food systems during the recovery period.

## 1. Introduction

Across the globe, the entire food supply chain has been disrupted by several preventive restrictions related to the COVID-19 pandemic, such as lockdowns, remote work, social distancing, etc., exposing its vulnerabilities to shocks and crises [1,2,3,4,5]. The Severe Acute Respiratory Syndrome Coronavirus 2 (SARS-CoV-2) has affected people’s lives and habits. Some of these behavioral and attitudinal shifts impacted food shopping [6,7], diet [8,9], and food safety attitudes [10], as well as food waste [11,12,13,14,15]. Further, the pandemic has amplified pre-existing economic weaknesses and brought new challenges by lowering incomes, as many people lost their jobs, and were faced with rising food prices and restricted food accessibility, which increased the challenge of achieving the Sustainable Development Goals (SDGs) [16]. In addition, the pandemic is revealing inequities between and within countries in terms of food security and economic opportunities. It significantly affects developing countries and fragile groups, deepening existing inequalities [17]. It has negatively impacted income and consumption, particularly among the poorest, who have lost jobs and have seen food prices rise [18].

Furthermore, the pandemic has varied consequences for men and women in various parts of the globe. Recent research has shown that women are disproportionately impacted by health and economic crises in various ways, including food security and nutrition, access to health facilities, services, and economic opportunities, and gender-based violence [19]. In addition, numerous studies have shown that the COVID-19 epidemic had a more significant impact on women than men [20,21,22,23,24]. While fatality rates are higher for men globally, the crisis’s socio-economic effects are particularly severe for women [19,25,26]. Women are more prone than males to work in low-wage, insecure sectors that are especially susceptible during recessions, generating another source of gender inequality [22,27,28]. Globally, women’s personal finances and employment security are weaker than men’s. In addition, women are more likely to be single parents, who will be struck harder by the current economic crisis [29]. Additionally, school and daycare facility closures have resulted in a tremendous rise in child care needs, disproportionately impacting working mothers [30].

Moreover, changes in routine, such as those in quarantine, were indicated to have more significant implications for women since they generally had more responsibility in family food decisions [31]. In addition, under stressful circumstances, women tend to eat more than males [31]. In addition, following an emotional eating episode, men were less likely to experience guilt than women [32]. Overall, the COVID-19 pandemic had a more significant impact on female households’ diet diversity than male households [33,34]. It has led to significant disturbances in women’s daily routines that may have an unexpected effect on eating habits [35]. During the pandemic, women in several countries were less likely to continue shopping as usual [36].

As seen worldwide, the COVID-19 pandemic-related measures in the Near East and North Africa (NENA) region have resulted in many lifestyle modifications, including changes in diet and food buying patterns among adults [13,37,38,39]. Numerous earlier studies underlined that social distancing measures applied across the region to contain the spread of the virus transformed lifestyle behaviors, including eating and food shopping habits, e.g., a surge in stockpiling [39] and online shopping [40,41]. Further, COVID-19 has forced people in the region to re-evaluate their lifestyles, and many have become more conscious about their diet. In order to improve their immune system to combat SARS-CoV-2, health has become a concern for individuals across the region [13]. During the COVID-19 pandemic, documented unhealthy eating behaviors and food choices included overeating, snacking, substituting main meals for snacks, increasing usage of delivery services, and highly processed food consumption.

Specifically, the pandemic impacted women and men differently in this region and exacerbated existing restrictive societal, religious, and cultural barriers and gender inequalities [42]. The NENA region has meager female labor force participation (FLFP) levels, the lowest globally, projected at 19.77% in 2019 [43]. Although gender disparity is a global issue, particular conservative social norms and legislative frameworks exacerbate women’s difficulties in the region [25]. Hence, according to the World Economic Forum’s Global Gender Gap Index, in 2020, the region has only closed 61.1% of its gender gap, which is the gap between men and women across health, education, politics, and economic opportunities [44]. Accordingly, the region is the least advanced geographical location regarding women’s equality [44]. For example, in Tunisia, which is depicted as a pioneer of women’s rights in the region, rural women remain socially and economically marginalized. Although they make up 70% of the Tunisian agricultural workforce, they are paid 50% less than males and have extremely little access to social security. While their employment circumstances were already dreadful, the pandemic has exacerbated their economic and social vulnerability [45].

Further, previous crises, such as the Arab Spring, proved disastrous for women’s rights in the region, as individuals took advantage of a crisis to reverse social progress—a trend currently being repeated during COVID-19 [46]. Accordingly, it is crucial to diagnose the extent to which the COVID-19 pandemic affected women and men’s food behaviors in the MENA region differently. However, data about the impact of the COVID-19 pandemic on women’s diets and food-related behaviors in the NENA region are scarce. Firstly, the academic research on the impacts of the COVID-19 pandemic on food systems and diet has been unevenly distributed across regions, with most studies focusing on Western and Southern Europe, North America, and China [47]. In contrast, developing countries in general, particularly those in the NENA region, have been neglected. Secondly, most of the existent studies on the impact of the pandemic on food activities in the NENA region focused on the general population [13,37,38,48,49,50,51] or students [10], and rarely on women.

Consequently, the purpose of this paper is to examine the impacts of the COVID-19 pandemic on women’s food habits and practices in three North African countries: Egypt, Morocco, and Tunisia. Accordingly, the present paper sought to achieve two major objectives: (1) to investigate how the COVID-19 pandemic impacted women’s diets; and (2) to investigate how the pandemic impacted women’s food shopping behaviors in North Africa. We outline the research methodology (Section 2) before presenting the study findings (Section 3), then discussing them and drawing the main conclusions (Section 4).

## 2. Materials and Methods

The paper is based on the results of an online survey conducted using the SurveyMonkey platform from 15 September to 5 November 2020. The survey was available in Arabic and French languages. The total number of valid collected responses was 995:343 in Egypt, 340 in Morocco, and 312 in Tunisia. The sample included 511 women: 212, 155, and 144 in each country, respectively.

The survey link was disseminated through social media, particularly Facebook, the most utilized social media in the region [52]. The research addresses the broad population of adults (>18 years of age) in the surveyed countries. The snowball sampling method was used, and participants were encouraged to share the online survey with their friends and family. We also adopted a nonprobability sample approach, in which survey respondents were selected at random and without regard for any previous characteristic other than age. Furthermore, there was no monetary remuneration for taking part in the study. The study was carried out in accordance with the Helsinki Declaration’s principles, and all methods involving research subjects were approved by the Western Michigan University Human Subjects Institutional Review Board (HSIRB) [53]. Before taking part in the research, all participants provided digital informed consent for data sharing and privacy policy.

Twenty-five different types of questions (both multiple-choice and one-option) were included in the questionnaire, separated into three sections (Appendix A). The first section included ten questions about the respondents’ socio-demographic characteristics (e.g., education level, gender, revenue, etc.). The second section included thirteen questions on their food buying behavior and diet (e.g., food shopping habits, nutrition activities, food waste, etc.). The third section included two questions on their moods and emotions during the pandemic. Prior to its distribution, the questionnaire underwent a two-phase evaluation. Firstly, to ensure the reliability of the study, an expert panel conducted a quality review of the content’s validity. Secondly, the questionnaire was pretested by 20 respondents in each country to verify the validity and reliability of the survey results.

The survey findings were downloaded from the SurveyMonkey platform into SPSS (Statistical Package for Social Sciences) version 25.0. We computed descriptive statistics (means, percentages, and frequencies). The percentages of answers and instances were calculated using multiple responses. Non-parametric tests were utilized since the variables were categorical and ordinal. The Mann–Whitney U test evaluated dichotomous, categorical independent variables. The Mann–Whitney U test allowed evaluating the effects of gender, a dichotomous variable (male/female), on food behaviors during the pandemic, and this resulted central in determining the main findings of the research, while the Kruskal–Wallis test assessed multi-choice replies (e.g., age and income). The Kruskal–Wallis test allowed assessing how the effects of the COVID-19 pandemic on food behaviors have been moderated by different variables such as age and income. The *p*-value for statistical significance was fixed at 0.05 for all tests.

## 3. Results

The survey results reveal that the COVID-19 pandemic has significantly influenced diets and food procurement, preparation, and usage in the three countries evaluated. We begin by presenting the socio-demographic features of the survey participants (*3.1*) and then investigate the gender-differentiated influence of the pandemic on consumption patterns (*3.2*) and food shopping (*3.3*).

### 3.1. Socio-Demographic Characteristics of the Survey Participants

Table 1 provides an overview of the respondents’ socio-economic background. According to the results, 51.4% were female, with significant variation in the countries studied. For instance, 68% of the Tunisian respondents were women, compared to 42% for Egypt and 45.6% for Morocco. In addition, 42.4% of the respondents were married with children, and 36.9% lived with their parents. Most respondents were middle-aged (58% were between 25 and 45), and 51.1% had a higher income than the other households in their respective countries. With 55.4% holding a master’s or doctoral degree or more, the sample was generally well educated. Only 4% of those polled had a secondary school diploma or no education credentials. In terms of occupation, 66.5% were employed (either full-time or part-time), 17.2% were students, and 8% were jobless and/or searching for a job (Table 1).

### 3.2. Food Consumption Patterns in the Context of the COVID-19 Pandemic

The data show that during the COVID-19 pandemic, there were substantial changes in consumer eating behavior and consumption patterns in the three countries analyzed. Firstly, as shown in Table 2, 9.43% of the cohort reported eating more food due to fear, anxiety, or boredom, whereas 70.56% did not. In Egypt, 27.70% of the respondents ate more food due to fear, anxiety, or boredom, while 30.97% and 39.62% did the same in Morocco and Tunisia, respectively. However, in Morocco, women ate more food due to fear, anxiety, or boredom than men (30.97% compared to 12.97%). The Mann–Whitney U test confirmed these findings, as shown in Table 3. Indeed, gender significantly influenced eating more food due to fear, anxiety, or boredom in Morocco. 

Secondly, 41% of the participants reported consuming more comfort food (e.g., candy, cookies, cakes, and pastries) (by including both “much more” and “moderately more” answers). In Egypt, Morocco, and Tunisia, 46.23%, 41.21%, and 35.57%, respectively, did the same. In general, women ate more comfort food than men, 43.91% compared to 36.34%. However, we discovered some disparities amongst the analyzed countries. In Morocco and Tunisia, women ate more comfort food than men (51.61% compared to 30.81% in Morocco and 39.15% compared to 32% in Tunisia). Meanwhile, in Egypt, men ate more comfort food than women, 46.23% compared to 40.98% (Table 2). According to Table 3, gender significantly influenced comfort food consumption in Morocco and Tunisia.

Thirdly, by counting “slightly less” and “much less”, 63.12% of the respondents decreased their consumption of unhealthy foods, such as fast food (Table 2). Only 20.06% of the cohort ate more of these food items. However, gender significantly influenced the consumption of these items, especially in Egypt and Morocco (Table 3). In fact, in Egypt, 25.70% of women increased their consumption of unhealthy foods compared to 11.05% for men. Meanwhile, in Morocco, 34.05% of men increased their consumption of unhealthy foods compared to 21.94% for women (Table 2).

### 3.3. Food Purchasing Habits during the COVID-19 Pandemic

As found in several countries globally [54,55], COVID-19 significantly impacted most participants’ food shopping and buying behaviors in the three studied countries. People’s purchasing habits have shifted since going to the supermarket in person is associated with a higher level of risk and a corresponding increase in anxiety about being close to people, despite the numerous precautions implemented by supermarkets. As a result, most participants decreased the number of shopping visits and shopped less than usual, purchasing more on each trip to minimize store visits and lower their perceived risk of COVID-19 exposure.

Indeed, according to Table 4, 62.8% of the participants stated that they shop less frequently than usual, while 9.9% mentioned they shop more frequently than usual. However, we observed some differences between the studied countries. While in Egypt and Tunisia, 70% of the participants indicated that they go shopping less than usual, only 54.7% did the same in Morocco. From a gender perspective, we did not observe differences between men and women in shopping behavior.

Meanwhile, as observed in Table 5, 32.42% of the participants indicated that they buy more than usual on each trip to the grocery store (by counting “a lot more” and “more”), and 48.45% did not change their behavior. The results indicate some differences between the studied countries as well. Indeed, in Tunisia and Morocco, 37.5% and 35.3% of the participants, respectively, indicated that they buy more than usual on each trip to the grocery store (by counting “a lot more” and “more”), but only 24.5% did so in Egypt.

These differences could be related to the stocking behaviors. Indeed, according to Table 6, there are some differences between the countries. In total, 49.94% of the cohort stocked food. In Tunisia and Morocco, 59.29% and 52.65% did so, respectively. However, only 37.90% of the Egyptian participants stocked food. As confirmed by the Mann–Whitney U test, gender influenced stocking up food in Egypt (Table 7); men stocked more food than women, 41.21% compared to 33.33%.

## 4. Discussion and Conclusions

In this paper, we evaluated the effects of COVID-19 on consumers’ food behaviors in three countries of the North Africa sub-region, namely, Egypt, Morocco, and Tunisia, with a specific focus on women. We witnessed a significant change in people’s attitudes and behaviors toward food and health, especially women, as they spent more time at home. Indeed, there have been noticeable changes in the way consumers eat, purchase, and engage with food. The study identified many significant consumer trends that are presently influencing food and health behaviors in the region.

Firstly, people experienced negative feelings, fear, and anxiety due to the pandemic. These unpleasant feelings led to overeating. Gender significantly influenced this behavior, especially in Morocco.

Secondly, the results indicated a shift toward a healthier diet during the COVID-19 pandemic. Despite the gravity of the issue, consumers may have experienced some unanticipated repercussions due to spending more time at home [56]. Indeed, most participants reported a reduction in their consumption of unhealthy foods such as sweets and fast food throughout the pandemic. In general, individuals in the region have been concerned about their health in order to strengthen their immune systems to combat SARS-CoV-2 [13]. The change from the pre-COVID-19 state towards healthier diet habits has been positive. However, gender significantly influenced this behavior, especially in Egypt, where women consumed more unhealthy food than men. The three studied countries have experienced rapid socio-economic development with demographic and lifestyle transitions in the past fifty years. Therefore, the prevalence of over-nutrition and associated morbidities grows. According to Seyfert et al. [57], a “nutrition transition” has occurred in the NENA region where traditional diets based mostly on food rich in fiber, vitamins, and micronutrients, such as grains and legumes, have been replaced by more contemporary, Westernized eating patterns that include more saturated fat, sugar, and processed foods. Coats et al. [58] pointed out that the NENA area is suffering from a double burden of malnutrition, with a high prevalence of undernutrition and growing rates of overweight and obesity, and resultant diet-related chronic diseases. Meanwhile, several micronutrient deficiencies (e.g., iron, iodine, zinc, calcium, folic acid, and vitamins A and D) continue to be recorded in several countries of the region, especially among susceptible populations, such as children and women [59]. The incidence of obesity in Egypt, for example, is among the highest in the world [60]. Indeed, the incidence of obesity among Egyptian adults reached 40% in 2019 [61]. Female Egyptians are more likely to be obese than males (about 50% compared to 30%, respectively). In Egypt, women are less likely to participate in physical activities than men due to cultural reasons [61,62]. Consequently, obesity is responsible for roughly three-quarters of all instances of adult type 2 diabetes in Egypt [60].

Thirdly, the pandemic has changed people’s shopping patterns since supermarkets are perceived as dangerous places where people are frightened to contact one other. COVID-19 was associated with fewer shopping visits and higher purchases per trip in the three studied countries. Furthermore, the results indicate a surge of stockpiling and panic buying of non-perishable food items, especially in Tunisia and Morocco. El Bilali et al. [51] found that 52.65% of participants had stockpiled food when COVID-19 became serious in Morocco. Indeed, there was a rush to supermarkets in Morocco just before the confinement in March 2020, and demand for flour and cereals skyrocketed. Moroccans were concerned about the coronavirus and were hoarding vast quantities. Food prices have soared as a consequence [63].

To the best of our knowledge, this is the first study of its kind in North Africa on the perceptions of the implications of COVID-19 on women’s food-related behaviors, and it lays the groundwork for future research into the pandemic’s gender-differentiated impacts. According to the results of this article, the long-term effects of COVID-19 will most likely vary not only from country to country, based on the socio-economic situation and shock resilience at the time of the outbreak [2], but also between women and men even within the same country.

Some limitations of the survey method and tool affect sample representativeness. As a result, the sample bias is the primary limitation of this study. The cohort was selected at random and recruited on a purely voluntary basis. The survey was conducted as a self-administered questionnaire by volunteers who were not compensated, and only individuals motivated by a serious interest in the topic participated in the research (cf. self-selection of the sample). Individuals with a high level of education, for example, were over-represented in our sample. Surveys often neglect individuals with a low level of education [64]. Individuals who are not web-literate, as well as the elderly, poor households, and informal laborers, are also excluded from online surveys, particularly in the NENA area. The abovementioned limitations are typical in computer-assisted web interviewing (CAWI), which is often used in surveys [65,66,67]. Because of this bias, it is challenging to extrapolate the poll results to the whole population in the studied countries. However, because of the COVID-19 pandemic, online surveys may gather data remotely, which is a significant advantage when social distance is required, and face-to-face interviews are challenging and hazardous.

This and other future studies will serve as a foundation for organizational and governmental readiness for future shocks and pandemic occurrences. Although the current research focused on the pandemic’s immediate and short-term consequences, larger and more representative samples (by including elderly, poor households, informal laborers, etc.) are required in future studies to understand better the virus’s medium- and long-term effects on food behaviors, such as food shopping and sourcing, diet, preparation, and food waste. For that, longitudinal studies are needed to grasp the evolution of the impacts of the pandemic over time and consequently inform the mitigation and recovery policies. Furthermore, future studies might concentrate on each SDG connected to the specifics of the food system, especially SDG 2 “Zero Hunger”, SDG 3 “Good health and wellbeing”, SDG 5 “Gender equality” and SDG 12 “Responsible consumption and production”.

## Figures and Tables

**Table 1:** Socio-demographic characteristics of the study participants (n = 995).

Variable	Item	Egypt (n = 343)	Morocco (n = 340)	Tunisia (n = 312)	Total (n = 995)
		%	%	%	%
**Gender**	Male	58	54.4	32.1	48.6
	Female	42	45.6	67.9	51.4
**Age**	18–24	33.5	28.3	6.4	23.2
	25–34	47.3	22.6	38.8	36.2
	35–44	10.2	23.5	32.7	21.8
	45-54	5.8	8.2	14.1	9.2
	>55	3.2	17.4	8.0	9.5
**Level of** **education**	No formal schooling		0.3		0.1
	Primary school				0.0
	Preparatory	0.6	1.2	0.3	0.7
	Secondary school	5.8	1.2	2.6	3.2
	University degree	67.1	21.2	32.7	40.6
	Higher degree (MSc or PhD)	26.5	76.2	64.4	55.4
**Income** **compared**	Much lower than most other households	2.9	8.5	11.9	7.6
	Slightly lower than most other households	7.0	7.6	9.9	8.1
	About the same as most other households	69.4	39.4	43.6	51.1
	Slightly higher than other households	19.5	44.4	34.6	32.8
	Much higher than other households	1.2	0.0	0.0	0.4
**Occupation**	In paid work (full time or part time)	65.3	63.8	70.8	66.5
	Student	18.4	20.3	12.5	17.2
	Unemployed and looking for work	9.9	5.6	10.3	8.5
	Home duties	4.4	0.9	2.9	2.7
	Retired/age pensioner	2.0	9.4	3.5	5.0
**Household composition**	Single person household	3.2	12.9	6.7	7.6
	Living with parents	46.6	31.5	32.1	36.9
	Married with children	38.5	41.8	47.4	42.4
	Married without children	2.6	8.2	7.4	6.0
	Extended family	8.7	4.7	3.8	5.8
	Shared household, non-related	0.3	0.9	2.6	1.2
**Losing Job**	Yes	32.1	13.2	16.7	20.8
	No	67.9	86.8	83.3	79.2

**Table 2:** Food eating behaviors during the COVID-19 pandemic (n = 995).

		Egypt (n = 343)			Morocco (n = 340)			Tunisia (n = 312)			Total (n = 995)		
		M	W	T	M	W	T	M	W	T	M	W	T
Eating more food out of fear, anxiety or boredom	Yes	28.14	27.08	27.70	12.97	30.97	21.18	39	39.62	39.42	26.71	32.55	29.43
	No	71.86	72.92	72.30	87.03	69.03	78.82	61	60.38	60.58	73.29	67.45	70.56
	Total	100	100	100	100	100	100	100	100	100	100	100	100
Eating comfort food	Less	10.56	6.94	10.56	4.86	6.45	5.65	9	6.13	7.56	8.14	6.50	7.92
	More	46.23	40.98	46.23	30.82	51.61	41.21	32	39.15	35.57	36.34	43.91	41
	Same	43.21	52.08	43.21	64.32	41.29	52.80	59	53.30	56.15	55.51	48.89	50.72
	Total	100	100	100	100	100	100	100	100	100	100	100	100
Eating unhealthy food	Less	63.82	47.22	55.52	52.43	65.16	58.79	73.12	72.75	63.12	61.71	62.41	63.12
	More	11.05	25.70	18.37	34.05	21.94	28	15.10	14.42	20.06	20.68	20.37	20.06
	Same	25.13	27.08	26.10	13.51%	12.90	13.24%	13.51	12.90	12.92	13.36	13.15	12.92
	Total	100	100	100	100	100	100	100	100	100	100	100	100

Legend: M: men, W: women, T: total.

**Table 3:** Effects of gender on some food-related behaviors.

Variables	Gender	Egypt (n = 343)		Morocco (n = 340)		Tunisia (n = 312)		Total (n = 995)	
		Mean Rank	U	Mean Rank	U	Mean Rank	U	Mean Rank	U
Eating more food out of fear, anxiety, or boredom	Male	179.13	12,908.5	184.45	11,757.5 **	162.38	10,012.0	528.74	108,784.5 **
	Female	162.14		153.85		153.73		468.89	
Eating more comfort food	Male	164.32	12,799.5	157.34	11,902.5 **	135.95	8544.5 **	462.87	106,657.5 **
	Female	182.61		186.21		166.20		531.28	
Eating more unhealthy food	Male	162.71	12,480.0 *	152.65	11,034.5 **	158.34	10,416.0	478.96	114,446.5 *
	Female	184.83		191.81		155.63		516.03	

Note: ** Statistically significant at *p*-value < 0.01, * Statistically significant at *p*-value < 0.05

**Table 4:** Change in shopping behavior (percentage) (n = 995).

	Egypt (n = 343)			Morocco (n = 340)			Tunisia (n = 312)			Total (n = 995)		
	M	W	T	M	W	T	M	W	T	M	W	T
**I go shopping less than usual**	69.35	70.83	70	55.68	53.55	54.7	59	66	70	62	63.6	62.8
**I go shopping like I used to**	29.65	26.39	28.28	32.43	27.10	30	27	21	28.3	30.16	24.5	27.2
**I go shopping more than usual**	1.01	2.78	1.75	11.89	19.35	15.3	14	13	1.7	7.85	12	9.9
**Total**	100	100	100	100	100	100	100	100	100	100	100	100

Legend: M: men, W: women, T: total.

**Table 5:** Change in food purchase behavior (percentage) (n = 995).

	Egypt (n = 343)			Morocco (n = 340)			Tunisia (n = 312)			Total (n = 995)		
	M	W	T	M	W	T	M	W	T	M	W	T
**I buy a lot more than usual**	7.0	7.64	7.3	8.11	10.32	9.12	8.00	8.49	8.33	7.71	8.81	8.24
**I buy more than usual**	18.59	15.28	17.20	25.41	27.10	26.18	36	26	29.17	26.66	22.8	24.18
**I buy as same as usual**	50.25	52.78	51.31	51.89	44.52	48.53	44	46.23	45.51	48.71	47.84	48.45
**I buy less than usual**	22.11	19.44	21	13.51	16.13	14.71	8	15.09	12.82	14.54	16.88	16.17
**I buy a lot less than usual**	2.01	4.86	3.21	1.08	1.94	1.47	4	4.25	4.17	2.36	3.68	3
**Total**	100	100	100	100	100	100	100	100	100	100	100	100

Legend: M: men, W: women, T: total.

**Table 6:** Food stockpiling behavior (percentage) (n = 995).

	Egypt (n = 343)			Morocco (n = 340)			Tunisia (n = 312)			Total (n = 995)		
	M	W	T	M	W	T	M	W	T	M	W	T
**Yes**	41.21	33.33	37.90	52.43	52.90	52.65	58	59.91	59.29	50.54	48.71	49.94
**No**	58.79	66.67	62.10	47.57	47.10	47.35	42	40.09	40.71	49.45	51.28	50.05
**Total**	100	100	100	100	100	100	100	100	100	100	100	100

Legend: M: men, W: women, T: total.

**Table 7:** Effects of gender on some food shopping behaviors (n = 995).

Variables	Gender	Egypt (n = 343)		Morocco (n = 340)		Tunisia (n = 312)		Total (n = 995)	
		Mean Rank	U	Mean Rank	U	Mean Rank	U	Mean Rank	U
Stockpiling more food	Male	173.09	14,111.5	164.42	13,213.5	150.86	10,036.0	475.21	112,629.5
	Female	170.50		177.75		159.16		519.59	
Change in shopping behavior	Male	166.33	13,200.0	170.86	14,270.0	158.52	10,398.0	501.39	122,021.5
	Female	179.83		170.06		155.55		494.79	

Legend: M: Men, W: Women, T: Total.

## Data Availability

The data used during the present investigation are available upon request to the corresponding author.

## Appendix A. Questionnaire

**Section 1: Socio-demographics**
1. Gender
2. Where do you live? Urban region/Rural area
3. Age
4. Level of education
5. How would you describe your household income compared to other households in your country?
6. Occupation
7. What is your household composition?
8. How many people are currently living in your home?
9. Have you lost your job or had any pay reduction in your salary due to COVID-19? Yes/No
10. Please indicate if any of people you know have been diagnosed with the coronavirus (COVID-19)?
**Section 2: Food buying and consumption behavior**
11. Below is a list of food-related behaviors. Please indicate how that behavior has changed for you as a result of the coronavirus (COVID-19) becoming serious in your country. (7-point response scale: never = 0; first time = 1; much less = 2; slightly less = 3; about the same = 4; moderately more = 5; much more = 6) ● Buying local food (produced in your country) ● Ordering groceries online ● Buying food in person from a large supermarket ● Buying food in person from a small supermarket or grocery store ● Having meals delivered directly to my home
12. What has changed in your shopping behavior during the outbreak of COVID-19 and lockdown? ● I go shopping less than usual ● I go shopping like I used to ● I go shopping more than usual
13. What has changed in the extent of your purchases during the outbreak of COVID-19 and lockdown? ● I buy a lot more than usual ● I buy more than usual ● I buy as same as usual ● I buy less than usual ● I buy a lot less than usual
14. Since the coronavirus (COVID-19) became serious in your country, have you eaten or drunk more or less of the following foods? (7-point response scale) ● Fruits/Vegetables ● Meat ● Healthy food ● Unhealthy food (fast food) ● Water ● Candy, cookies, cakes, and pastries ● Healthy snacks ● Unhealthy snacks ● Packaged frozen foods ● Canned food
15. Since the coronavirus (COVID-19) became serious in your country, have you done more or less of the following food-related activities than you used to? (7-point response scale) ● Eating at home alone ● Eating with family members ● Eating out (e.g., restaurants/cafeteria/fast food) ● Eating at someone else’s place (e.g., family, friends) ● Ordering take-away or fast-food meals with deliveries ● Cooking and preparing food ● Spending a lot of time cooking ● Making easy meals (e.g., instant foods, etc.) ● Eating between meals (e.g., snacks)
16. Have you stocked up on food and beverages because of the coronavirus (COVID-19)? Yes/No
17. What type of food did you stock up the most during the outbreak of COVID-19 and lockdown? (Please select all that apply) ● Cereals and their products (bread, rice, pasta, flour, etc.) ● Roots and tubers (potatoes, etc.) ● Legumes (e.g., peas, chickpeas) ● Sugar ● Oil ● Fruits and vegetables ● Meat and meat products ● Fish and seafood ● Milk and dairy products ● Canned food ● None
18. Since the COVID-19 outbreak, did you notice that any of these items are less available? (Please select all that apply) ● Cereals and products (bread, rice, pasta, flour, etc.) ● Roots and tubers (potatoes, etc.) ● Legumes (e.g., peas, chickpeas) ● Sugar ● Oil ● Fruits and vegetables ● Meat and meat products ● Fish and seafood ● Milk and dairy products ● Canned food ● None
19. Since the COVID-19 outbreak, did you notice any price increase for any of these items? (Please select all that apply) ● Cereals and products (bread, rice, pasta, flour, etc.) ● Roots and tubers (potatoes, etc.) ● Legumes (e.g., peas, chickpeas) ● Sugar ● Oil ● Fruits and vegetables ● Meat and meat products ● Fish and seafood ● Milk and dairy products ● Canned food ● None
20. How does stocking up on items make you feel? (5-point response scale: 1 (not at all), 5 (very much)) ● Stocking up on items makes me feel less anxious ● Stocking up on items makes me feel more secure ● Stocking up on items comforts me ● Stocking up on items gives me a sense of control
21. Please indicate how concerned you have been since COVID-19 became serious in your country about the following food-related issues. (5-point response scale: 1 (not at all), 5 (very much)) ● Obtaining enough food ● Obtaining a variety of food ● Access to healthy and safe food ● Food prices rising ● Food spreading COVID-19
22. Regarding changes in your food-related behaviors since the outbreak of COVID-19: Yes/No ● Do you buy more food out of fear or anxiety? ● Do you eat more food out of fear, anxiety or boredom? ● Are you wasting more food than usual? ● Are you more aware of how much food you waste?
23. How has your food wastage changed during the outbreak of COVID-19 and lockdown? ● It has become much less ● Less ● Has not changed ● More ● Much more
**Section 3: Emotions**
24. Please indicate your negative feelings since the onset of COVID-19. (5-point response scale: 1 (not at all), 5 (very much)) ● Nervous ● Worried ● Depressed ● Sad ● Scared ● Bored
25. Please indicate your positive feelings since the onset of COVID-19. (5-point response scale: 1 (not at all), 5 (very much)) ● Calm ● Optimistic ● Excited ● Happy
